# Anti-inflammatory and anti-invasive effects of *α*-melanocyte-stimulating hormone in human melanoma cells

**DOI:** 10.1038/sj.bjc.6601349

**Published:** 2003-11-11

**Authors:** P Eves, J Haycock, C Layton, M Wagner, H Kemp, M Szabo, R Morandini, G Ghanem, J C García-Borrón, C Jiménez-Cervantes, S Mac Neil

**Affiliations:** 1 University Section of Medicine, Division of Clinical Sciences, Northern General Hospital, Sheffield S5 7AU, UK; 2 Department of Engineering Materials, University of Sheffield, Mappin Street, Sheffield S1 3JD, UK; 3 Department of Histopathology, Northern General Hospital Trust, Sheffield S5 7AU, UK; 4 Laboratory of Oncology and Experimental Surgery, Institut Bordet, Université Libre de Bruxelles, Belgium; 5 Department of Biochemistry and Molecular Biology, School of Medicine, University of Murcia, Apto 4021, 30100 Espinardo, Murcia, Spain

**Keywords:** melanoma, *α*-MSH, melanocortin, NF-kappaB, metastasis

## Abstract

*α*-Melanocyte stimulating hormone (*α*-MSH) is known to have pleiotrophic functions including pigmentary, anti-inflammatory, antipyretic and immunoregulatory roles in the mammalian body. It is also reported to influence melanoma invasion with levels of *α*-, *β*- and *γ*-MSH correlated clinically with malignant melanoma development, but other studies suggest *α*-MSH acts to retard invasion. In the present study, we investigated the action of *α*-MSH on three human melanoma cell lines (HBL, A375-SM and C8161) differing in metastatic potential. *α*-melanocyte-simulating hormone reduced invasion through fibronectin and also through a human reconstructed skin composite model for the HBL line, and inhibited proinflammatory cytokine-stimulated activation of the NF-*κ*B transcription factor. However, A375-SM and C8161 cells did not respond to *α*-MSH. Immunofluorescent microscopy and Western blotting identified melanocortin-1 receptor (MC-1R) expression for all three lines and MC-2R on HBL and A375-SM lines. Receptor binding identified a similar affinity for *α*-MSH for all three lines with the highest number of binding sites on HBL cells. Only the HBL melanoma line demonstrated a detectable cyclic adenosine monophosphate (cAMP) response to *α*-MSH, although all three lines responded to acute *α*-MSH addition (+(−)-*N*^6^-(2-phenylisopropyl)-adenosine (PIA)) with an elevation in intracellular calcium. The nonresponsive lines displayed MC-1R polymorphisms (C8161, Arg (wt) 151/Cys 151; A375-SM, homozygous Cys 151), whereas the HBL line was wild type. Stable transfection of the C8161 line with wild-type MC-1R produced cells whose invasion was significantly inhibited by *α*-MSH. From this data, we conclude that *α*-MSH can reduce melanoma cell invasion and protect cells against proinflammatory cytokine attack in cells with the wild-type receptor (HBL).

The aim of this study was to investigate how *α*-melanocyte-simulating hormone (α-MSH) affects melanoma cell invasion and resistance to proinflammatory cytokines. *α*-melanocyte-simulating hormone arises from the proteolytic cleavage of pro-opiomelanocortin (POMC) and is responsible for pigmentation in lower vertebrates (Eberle 1988). POMC and *α*-MSH are synthesised by normal human keratinocytes and melanocytes (Schauer *et al*, 1994; Kippenberger *et al*, 1995; Chakraborty *et al*, 1996), and administration *in vivo* can produce skin darkening in humans (Lerner and McGuire 1961). Although cultured mouse melanocytes and melanoma cells reliably pigment to *α*-MSH addition (Eberle, 1988), human melanocytes have little or no response (Friedmann *et al*, 1990; De Luca *et al*, 1993; Hedley *et al*, 1998a). Responses relate to skin type (Hunt *et al*, 1994) with melanocytes from type 1 and type 2 skin donors rarely pigmenting to *α*-MSH *in vitro*, whether in 2-D (Hedley *et al*, 1998a) or 3-D culture (Hedley *et al*, 2002).

*α*-Melanocyte-simulating hormone also has potent antipyretic and anti-inflammatory responses, inhibiting acute and chronic inflammation in a number of tissues (Lipton and Catania, 1997), including for example ultraviolet (UV) irradiation (Luger *et al*, 1999). Our group has showed that *α*-MSH can inhibit tumour necrosis factor-α (TNF-*α*)-induced upregulation of intercellular adhesion molecule-1 (ICAM-1) in human melanocytes and melanoma cells (Hedley *et al*, 1998b; Morandini *et al*, 1998) and reduce interactions between cytokine-stimulated melanoma cells and T lymphocytes (Hedley *et al*, 2000). The expression of inflammatory cytokines and adhesion receptors such as ICAM-1 are largely under the control of the transcription factor NF-*κ*B (Ghosh *et al*, 1998). We also found *α*-MSH to reduce TNF-*α*-stimulated NF-*κ*B activity (and oxidative stress) in keratinocyte and melanoma lines (Haycock *et al*, 1999, 2000), suggesting protection of the epidermis from inflammatory and oxidative stresses. In addition, B16 melanoma cells respond to *α*-MSH with inhibition of growth and reduced adhesion (Robinson and Healy, 2002).

Melanocortin (MC) action arises via G-protein receptor signalling. Five receptors (MC-1R–MC-5R) have been cloned to date (Mountjoy *et al*, 1992) and MC-1R is expressed on human keratinocytes, melanocytes and melanoma cells (Donatien *et al*, 1992; De Luca *et al*, 1993; Bohm *et al*, 1999). Loss-of-function polymorphic variants exist (Valverde *et al*, 1995) and are associated with red-haired individuals who pigment poorly (Schiöth *et al*, 1999). Reports also suggest that MC-1R polymorphisms correlate with cutaneous melanoma (Box *et al*, 1997; Jiménez-Cervantes *et al*, 2001), although not all studies have confirmed this (Ichii-Jones *et al*, 1998).

Melanoma patients show high levels of *α*-MSH in plasma and tumours (Ghanem *et al*, 1989a, 1989b), thought to correlate with malignant melanoma development (Liu and Johansson, 1995). However, *α*-MSH is reported to both stimulate and inhibit melanoma invasion *in vitro* and *in vivo*. Murine melanoma cells respond to *α*-MSH stimulation *in vitro* with a higher number of metastases *in vivo*, and a positive correlation exists between murine melanoma cell response to *α*-MSH (assessed by cAMP elevation) *in vitro* and metastatic success *in vivo* (Sheppard *et al*, 1984; Hill *et al*, 1990). However, *α*-MSH can inhibit B16-BL6 melanoma invasion through reconstituted (Matrigel) basement membrane (Murata *et al*, 1997), and reduce colony formation by 50%, in agreement with similar work in B16-F10 cells (Kameyama *et al*, 1990). Thus, it is unclear if *α*-MSH promotes or inhibits melanoma invasion, or if it is the immune response to melanoma.

As the role of *α*-MSH in melanoma progression is unclear, and in light of additional cytoprotective roles, the aim of this study was to investigate the actions of *α*-MSH on both melanoma cell invasion and the ability of *α*-MSH to attenuate the response of cells to proinflammatory cytokines using human melanoma lines.

## MATERIALS AND METHODS

### Materials

Dulbecco's modified Eagle's medium (DMEM), glutamine, penicillin, streptomycin, amphotericin B, vitamin concentrate, Triton X 100, newborn calf serum (NBCS), Ham's F10 and F12, nonessential amino acids (NEA) and trypsin/EDTA were from Gibco BRL, Paisley, UK. Dispase was from Difco Laboratories, Detroit, USA. Fetal calf serum (FCS) was from GlobePharm Ltd, Esher, UK. Phosphate-buffered saline (PBS) was from Oxoid Ltd, Basingstoke, UK. Transwell™ plates (24-well) were from Corning Costar Corporation, Cambridge, MA, USA. Collagenase A and TNF-*α* were from Boehringer Mannheim, Lewes, UK. Tri-iodothyramine, epidermal growth factor (EGF), hydrocortisone, adenine, insulin, transferrin, isobutyl methylxanthine (IBMX), MTT, *N*-(A-Rhamnopyranosyloxyhydroxy-phosphinyl)-leu-trp sodium (Phosphoramidon), RPMI-1640, sodium pyruvate, D-glucose, bovine serum albumin (BSA), Cell Dissociation Solution, human plasma fibronectin, (−)-*N*^6^-(2-phenylisopropyl)-adenosine (PIA) and trypan blue were from Sigma Chemicals Ltd, Poole, UK. Paraformaldehyde, NH_4_Cl, glycerol, circular glass coverslips and poly-L-lysine-coated microscope slides were from BDH, Poole, UK. *α*-Melanocyte-simulating hormone was from Bachem, Essex, UK. Primary goat polyclonal IgG-specific antibody to MC-1R (N-19) and MC-2R (C-16), and anti-NF-*κ*B (p65) goat polyclonal IgG were from Santa Cruz Biotechnology Inc., CA, USA. Biotinylated anti-goat IgG and Streptavidin-FITC were from Vector Laboratories, Burlingame, USA. Fura-2-AM was from CN Biosciences, Beeston, UK. Prolong Antifade coverslip mounting medium was from Molecular Probes, Leiden, the Netherlands. Murine anti-HMB45 IgM was from Dako, Carpintera, USA. Bicinchoninic acid (BCA) assay kit was from Pierce (Tattenhall, Cheshire, UK). Enhanced chemiluminescence (ECL) kit was from AmershamPharmacia (Amersham, UK). Wizard kit and TfX™-50 Reagent were from Promega (Southampton, UK). pRc/CMV and geneticin (G-418) were from Invitrogen (Groningen, The Netherlands). All chemicals were of analytical grade unless otherwise indicated.

## METHODS

### Keratinocyte cell culture

Normal human skin keratinocytes were isolated as detailed previously (Chakrabarty *et al*, 1999; Eves *et al*, 2000). Freshly isolated cell suspensions were seeded onto dermal composites at 1 × 10^6^ viable cells within a 1 cm^2^ stainless steel ring (see the section ‘Reconstructed skin model of invasion’ below for further details).

### Human fibroblast cell culture

Dermal layers obtained after keratinocyte isolation were then used to isolate human skin fibroblasts (detailed in Chakrabarty *et al*, (1999); Eves *et al*, 2000). Fibroblast cells (passage 3–passage 9) were seeded onto dermal composites at 1 × 10^5^ cells per composite ring (see the section ‘Reconstructed skin model of invasion’ below for further details).

### Human melanoma cell line culture

Three human cutaneous melanoma cell lines were used: (i) HBL; (ii) A375-SM and (iii) C8161. HBL is derived from a lymph node metastasis of a nodular melanoma established in one of our laboratories (Ghanem *et al*, 1988). Cells were maintained as detailed in Eves *et al* (2000) and passaging was in 0.02% EDTA. For composite experiments, HBLs were resuspended in keratinocyte culture medium (KCM); for invasion, attachment and proliferation assays, HBL cells were resuspended in serum-free invasion assay medium (SFIAM). For MC-1R and NF-*κ*B immunolabelling, and calcium and cyclic adenosine monophosphate (cAMP) experiments, HBLs were resuspended in Ham's F10 melanoma culture medium (HMCM). The A375-SM cell line was a generous gift from Professor IJ Fidler (USA) via Professor MJ Humphries (University of Manchester, UK). A375 was established in culture from a lymph node metastasis of a 54-year-old female (Giard *et al*, 1973). These cells are heterogeneous in nature and a highly metastatic variant (A375-SM) was established in culture from lung metastases produced by parental A375 cells growing subcutaneously in nude mice (Kozlowski *et al*, 1984). The C8161 melanoma line was kindly donated by Professor F Meyskens (University of California, Irvine, USA) via Professor M Edwards (University of Glasgow, UK). C8161 was established from an abdominal wall metastasis (Bregman and Meyskens, 1983). Both A375-SM and C8161 cells (and B16 F10C1 murine melanoma cells used as a positive control for measurement of cAMP) were cultured in Eagle's modified essential medium (EMEM) supplemented with 10% (v v^−1^) FCS, 2 *μ*M L-glutamine, 100 IU ml^−1^ penicillin and 100 *μ*g ml^−1^ streptomycin, 1.2 *μ*g ml^−1^ amphotericin B, 1.5% (v v^−1^) (100 × stock) vitamin concentrate, 1 × 10^−3^ mol l^−1^ sodium pyruvate, 1% (v v^−1^) NEA and 0.187% (v v^−1^) sodium hydrogen carbonate, and incubated at 37°C in humidified 5% carbon dioxide/95% air. When approximately 80–90% confluent, cells were passaged using Cell Dissociation Solution. For invasion, attachment and proliferation assays, A375-SM and C8161 melanoma cells were resuspended in SFIAM, and for MC-1 receptor and NF-*κ*B immunolabelling and calcium and cAMP experiments, cells were resuspended in complete EMEM.

### Generation of a melanoma cell line with stable expression of MC-1 receptor

A stable C8161 melanoma cell line expressing functional MC-1 receptors was isolated by transfecting C8161 cells with vector pRc/CMV carrying MC-1 cDNA (kindly donated by Professor JES Wikberg, Uppsala University, Sweden). Briefly, cells were plated in 100 mm dishes in complete EMEM media. After growth to approximately 70% confluence, the cells were transfected with 15 *μ*g of pRC/CMV/MC-1 DNA using Tfx™-50 Reagent, according to the manufacturer's (Promega) protocol. Individual transfectants were isolated and cloned by limiting dilution following growth in complete EMEM media containing 500 *μ*g ml^−1^ geneticin (G-418).

### Indirect immunofluorescent labelling of MC-1R and NF*κ*B/p65

C8161 (5 × 10^3^ cells ml^−1^) and A375-SM (1 × 10^4^  cells ml^−1^) cells were resuspended in EMEM, and HBL (3 × 10^4^ cells ml^−1^) were resuspended in HMCM and seeded onto sterile circular glass coverslips (13 mm diameter) in 24-well plates and incubated at 37°C in humidified 5% carbon dioxide/95% air until approximately 80–90% confluent. For the NF*κ*B/p65 studies, melanoma cells were incubated in the appropriate medium (HMCM/EMEM)±*α*-MSH (10^−12^10^−6^ M) for 15 min, followed by a 1 h incubation in fresh medium±TNF-*α* (300 U ml^−1^). Cells were immunofluorescently labelled for MC-1R and NF*κ*B/p65 as detailed previously (Moustafa *et al*, 2002). Melanocortin-1 receptors and NF*κ*B/p65 in each melanoma were visualised using epifluorescent illumination (FITC filter, *λ*_ex_=490 nm, *λ*_em_=523 nm), and nuclei were visualised using a rhodamine filter (*λ*_ex_=555 nm, *λ*_em_=580 nm) with a Nikon X600 microscope. Digital images of MC-1R/NF*κ*B/p65 and nuclei were captured and overlaid using Adobe Photoshop. Cellular movement of NF-*κ*B/p65 was evaluated as described previously (Moustafa *et al*, 2002).

### Western blotting for MC-1R and MC-2R

Melanoma cells were seeded into six-well plates at 10^5^ cells per well and grown until 60% confluent. Western blot analysis was carried out as detailed previously (Moustafa *et al*, 2002). Immunoreactive bands were visualized using an ECL kit.

### MSH receptor binding studies

Saturation and competitive binding assays were performed using the following modification of a previously published method (Ghanem *et al*, 1988). Saturation assays allowed calculation of total receptor numbers and competitive binding allowed calculation of the affinity of the receptors for the peptide. Briefly, 250 × 10^3^ d.p.m. of ^125^I-[Nle^4^, Dphe^7^]-*α*-MSH in 100 *μ*l was added to 10^6^ cells (100 *μ*l) previously mixed with [Nle^4^, Dphe^7^]-*α*-MSH (concentration ranging from 10^−12^ to 10^−6^ M) in PBS pH 7.2 containing 0.1% BSA, 6.25 mM Hepes and 0.1% trasylol. After mixing, the tubes were incubated at 37°C for 45 min before cell separation by repeated centrifugation and washing. The pellet-associated radioactivity was then measured in a gamma counter.

### DNA extraction, amplification and sequencing of MC-1R

Briefly, genomic DNA was extracted from the HBL, C8161 and A375-SM cell lines using a Wizard kit. The full-length coding sequence of the *MC-1R* gene was amplified using primers as detailed previously (Jiménez-Cervantes *et al*, 2001).

### Measurement of intracellular free calcium

Cells were grown as monolayers on glass coverslips until nearly confluent and incubated with 4 *μ*M Fura-2 AM for 15 min at 37°C. Cells were then washed with balanced salt solution (1.5 mM calcium chloride, 0.5 mM magnesium chloride, 135 mM sodium chloride, 4.5 mM potassium chloride, 5.6 mM glucose, 10 mM Hepes, pH 7.4), and coverslips of cells were examined in a fluorimeter (Kontron SKM 25) to determine changes in intracellular calcium in response to the adenosine agonist PIA (10 *μ*M), and *α*-MSH (10^−12^ M to 10^−6^ M) alone or in combination. Details are as previously described in Metcalfe *et al* (1998).

### Measurement of cAMP

Both intracellular and extracellular cAMP responses to peptide addition were examined. For intracellular cAMP, cells were prelabelled with tritiated adenosine for 2 h. Cells were then preincubated with 0.8 mM IBMX for 15 min prior to addition of *α*-MSH at a range of concentrations for 10 min. Cells were washed with PBS, 100 *μ*l ethanol was added and the cells were stored at −20°C. Assay details are as previously described (Metcalfe *et al*, 1998). Extracellular cAMP in the media was determined using a commercially available kit (Biomedical Technologies Inc., USA), in which cAMP measurement is based on competitive binding between cAMP and an alkaline phosphate derivative of cAMP for a limited amount of specific antibody. A secondary antibody separates the bound cAMP from the free cAMP. A coloured end product is determined using a microtitre plate reader, measuring absorbance at 405–410 nm. Results shown are of three separate experiments.

### Fibronectin invasion assay

Transwell™ inserts were coated with a layer of human fibronectin as described previously (Dewhurst *et al*, 1997), and placed onto a 24-well tissue culture plate containing 600 *μ*l SFIAM (RPMI-1640 medium supplemented with 20 ng ml^−1^ EGF, 0.2% (w v^−1^) D-glucose solution and 0.1% (w v^−1^) BSA). Cell suspensions (50 *μ*l containing 0.6 × 10^5^ A375-SM cells or 1.2 × 10^5^ HBL/C8161 cells in SFIAM) plus an equivalent volume of SFIAM (±pharmacological agent) were added to each insert and cells were incubated for 15–52 h at 37°C in 5% CO_2_/95% air. As cell lines invaded at different rates, HBL cells were cultured for 20 h (Dewhurst *et al*, 1997; Richardson *et al*, 1999), C8161 cells for 15 h and A375-SM cells for 52 h, to obtain comparable levels of invasion. For the 52 h incubation time, a cell density of 0.6 × 10^5^ cells was used. After incubation, medium (containing cells) was collected from the upper and lower compartments of the Transwell™ chamber and placed into preweighed LP4 perspex tubes. The medium removed was replaced with an equivalent volume of 0.1% (w v^−1^) trypsin and 0.02% (w v^−1^) EDTA and the cells were incubated (10 min, 37°C). Trypsin (plus cells) was removed and placed in sample-matched tubes and a second trypsin incubation, followed by gentle scraping of the polycarbonate filter surface (upper chamber) and the undersides of each polycarbonate filter as well as the tissue culture plastic of the lower chambers, ensured that all the cells were harvested. Cells were centrifuged (200 **g**, 5 min), the majority of supernatant removed and tubes reweighed. Cells from each tube were resuspended in the remaining medium and the cell numbers counted using a haemocytometer. Noninvading cells were those remaining in the Transwell™ upper chamber and those attached to the upper surface of the filter. Invading cells were those removed from the underside of the filter, suspended in the medium of the tissue culture wells and attached to the base of the 24-well chamber (cells collected into the lower tubes). A combined analysis of cell counts plus obtained weight/volume for each cell suspension (weight of tubes containing cell suspensions minus the weight of empty tubes) provided a percentage of the total population of cells that had invaded through fibronectin. (This ‘total housekeeping’ approach to counting all cells is an improvement on the assay first described by us (Dewhurst *et al*, 1997)).

### Reconstructed skin model of invasion

Full details have been described previously (Chakrabarty *et al*, 1999; Eves *et al*, 2000). Briefly, sterile human skin was rehydrated in PBS and incubated in 1 M NaCl, which resulted in a de-epidermised acellular dermis (DED) with retained basement membrane antigens. Dermal fibroblast cells (1 × 10^5^) were added to the reticular DED surface. After 48 h, HBL melanoma cells (5 × 10^4^) and epidermal keratinocytes (1 × 10^6^) were added to the papillary DED surface. HBL melanoma cells were seeded 4 h before keratinocytes. After 48 h, the reconstructed skin composites were raised to an air-medium interface containing *α*-MSH (1 × 10^−9^ M)/IBMX (50 *μ*M) or medium alone (control). Composites were cultured for 2 weeks and the culture medium was changed every 2–3 days (±*α*-MSH (1 × 10^−9^ M)/IBMX (50 *μ*M)). External appearance of the upper surface of the composites was documented by photography prior to fixing in formalin. Paraffin-fixed histology sections (10 *μ*m) were made for haematoxylin and eosin staining and for immunolabelling of HMB45 for the identification of HBL melanoma cells (previously described in Eves *et al*, 2000). To assess the extent of invasion, a semiquantitative scoring system of 0–3 was used. Sections through the centre of the 1 cm diameter composite were examined for invasion of HBL cells. In each case, a score of 0 (zero) represented composites where no melanoma cells were present in the dermis. A score of 0.5 represented 1–5 cells in the dermis, 1.0, 6–15 cells in the dermis. A score of 1.5 represented 16–20 cells in the dermis, 2.0, 21–30 cells in the dermis and 2.5, 31–40 cells in the dermis. A score of 3.0 was reserved for florid invasion into the dermis where there were too many cells to count (+50 cells).

### Statistics

Student's paired *t*-test was used to analyse data where identical cellular treatments were present as a continuous series and where the only variable involved was the concentration of peptide or drug used. Nonparametric Mann–Whitney *U*-test was used to assess differences in the invasion of melanoma cells in a skin model containing several variables.

## RESULTS

### Melanocortin-1 and MC-2 receptor expression

**Figure 1:**
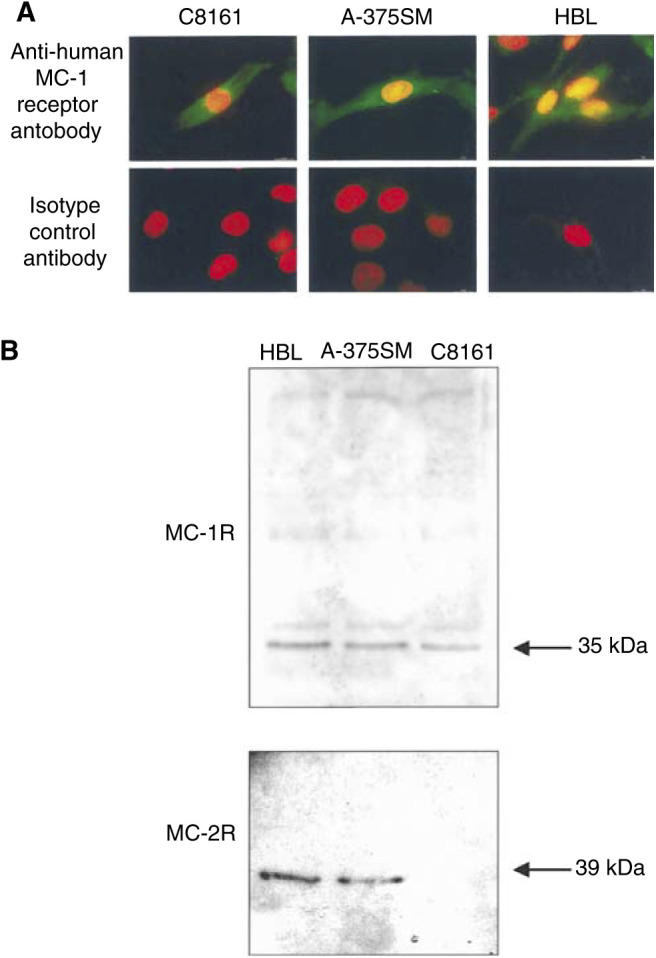
(**A**) Upper micrographs: Presence of MC-1 receptors on HBL, A375-SM and C8161 melanoma cell lines. Melanocortin-1 receptors (green fluorescence) were visualised using epifluorescent illumination (FITC filter, *λ*_ex_=490 nm, *λ*_em_=523 nm) and nuclei (red fluorescence) were visualised with a rhodamine filter (*λ*_ex_=555 nm, *λ*_em_=580 nm). Lower micrographs: Corresponding cell lines using isotype control primary antibody demonstrating observed specificity of immunolabelling. Bar=20 *μ*m. (**B**) Western blotting of HBL, A375-SM and C8161 melanoma cell lines for MC-1 and MC-2 receptors.

All three melanoma cells were observed to label positively, albeit weakly, for MC-1R as identified by immunofluorescent microscopy (Figure 1A

). HEK293 cells (which do not express MC-1R) did not label, as confirmed previously (Moustafa *et al*, 2002). No immunoreactivity was observed for cells incubated with isotype control serum. Western immunoblotting using the same primary anti-MC-1R antibody revealed a reactive band at 35 kDa molecular mass for the HBL, A375-SM and C8161 melanoma cell lines (Figure 1B); close to the predicted size of MC-1R, based on sequencing information (Mountjoy *et al*, 1992). The extracts of HEK293 cells did not reveal a similar band. Western blotting using a primary anti-MC-2R antibody revealed a reactive band at 39 kDa molecular mass for the HBL and A375-SM cell lines, but not in C8161 cells (Figure 1B). Higher molecular weight bands were also observed for both MC-1 and MC-2 receptors, indicating crossreactivity of the polyclonal sera used.

### Melanocortin receptor binding

**Figure 2:**
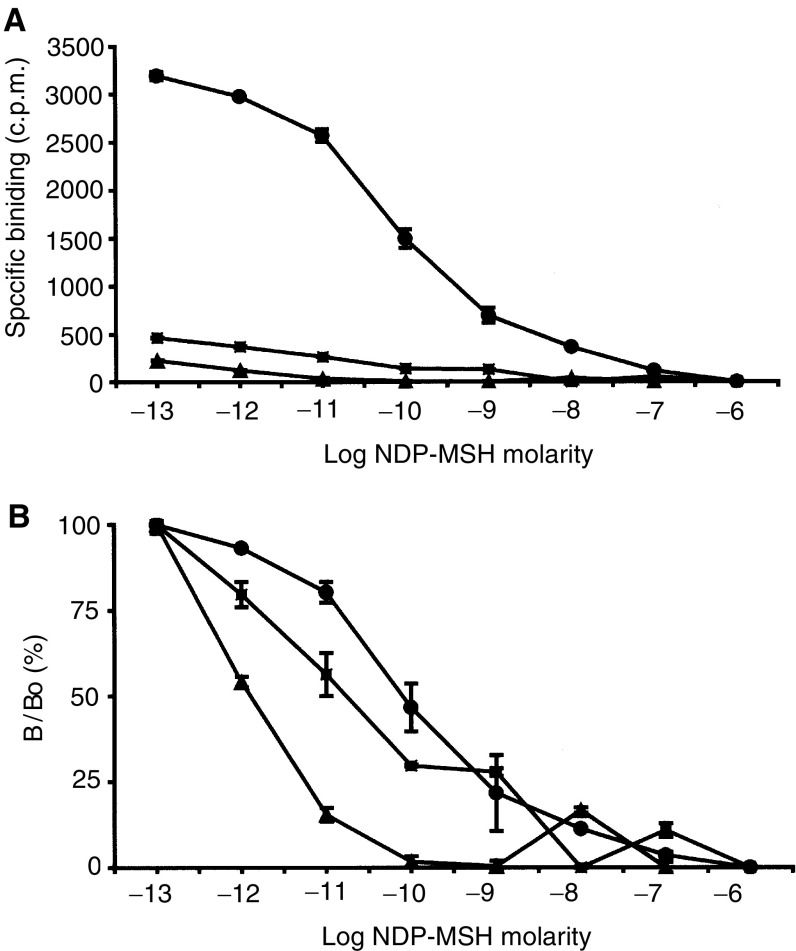
(**A**) Competitive binding of *α*-MSH for radiolabelled Nle4DPhe7-MSH was performed to saturation showing displacement data for HBL, A375-SM and C8161 melanoma cells, allowing calculation of total receptor numbers. (**B**) Competitive binding data was used to determine the affinity of melanoma cells for *α*-MSH (circles, HBL; squares, C8161; triangles, A375-SM; means±s.e.m., *n*=3).

Receptor saturation studies demonstrated that HBL melanoma cells (as previously noted) had around 1000–3000 binding sites per cell and C8161 cells had around 400 receptors per cell (Figure 2A and B

). The receptor number for the A375-SM cell line was much lower and not statistically different to the level of nonspecific binding achieved (Figure 2). The three cell lines demonstrated similar binding affinities for *α*-MSH (summarised in Figure 2B). However, caution must be used in considering the binding affinity for the A375-SM due to a relatively low number of receptors expressed per cell.

### Melanocortin-1 receptor sequences

Sequencing of the MC-1R genomic DNA revealed the HBL melanoma line to be homozygous wild type, the A375-SM line to contain a homozygous polymorphism for Arg151Cys, while the C8161 line was heterozygous for Arg151Cys, with the other allele displaying a wild type sequence.

### *α*-Melanocyte stimulating hormone, cAMP and intracellular calcium

**Figure 3:**
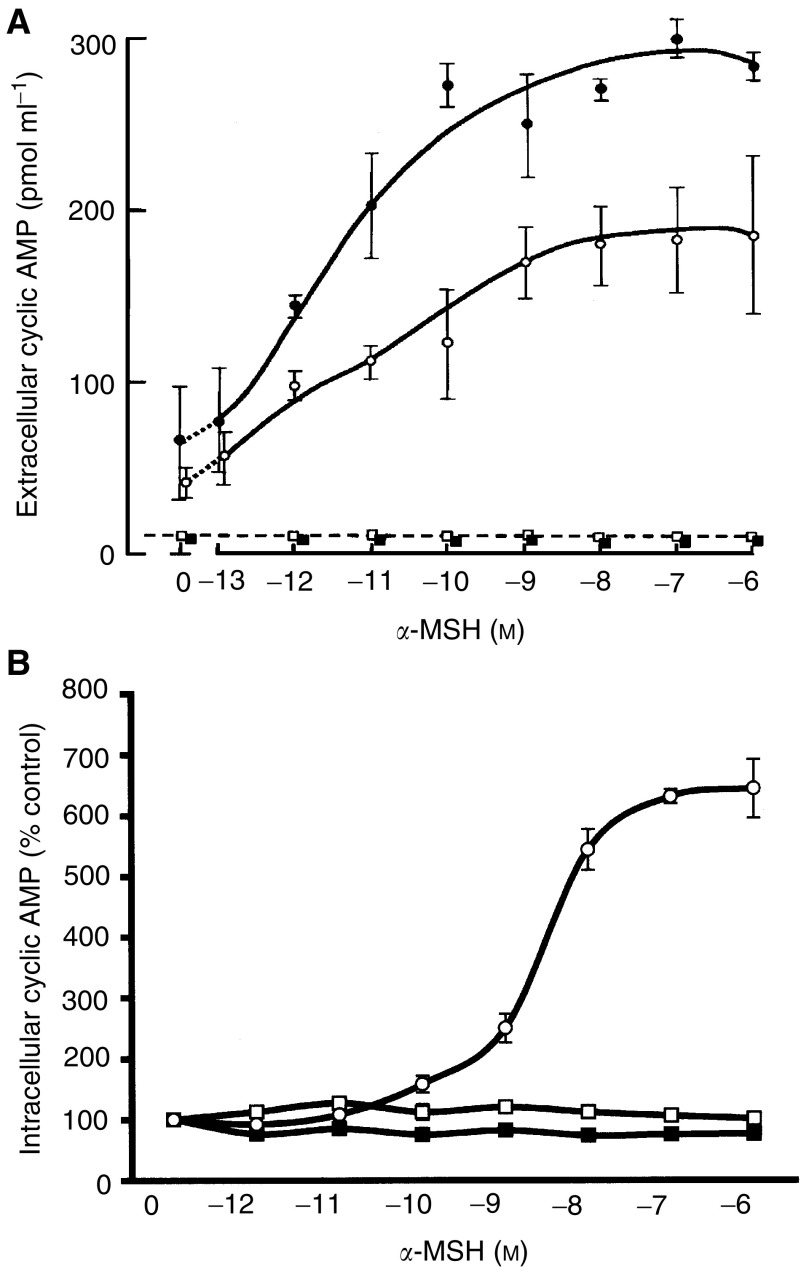
Detection of extracellular (**A**) and intracellular (**B**) cAMP in response to increasing concentrations of *α*-MSH. Symbols: filled circles, mouse melanoma B16F10C1 (positive control); open circles, HBL melanoma cells; filled squares, A375-SM melanoma cells; open squares, C8161 melanoma cells (means±s.e.m., *n*=3).

**Figure 4:**
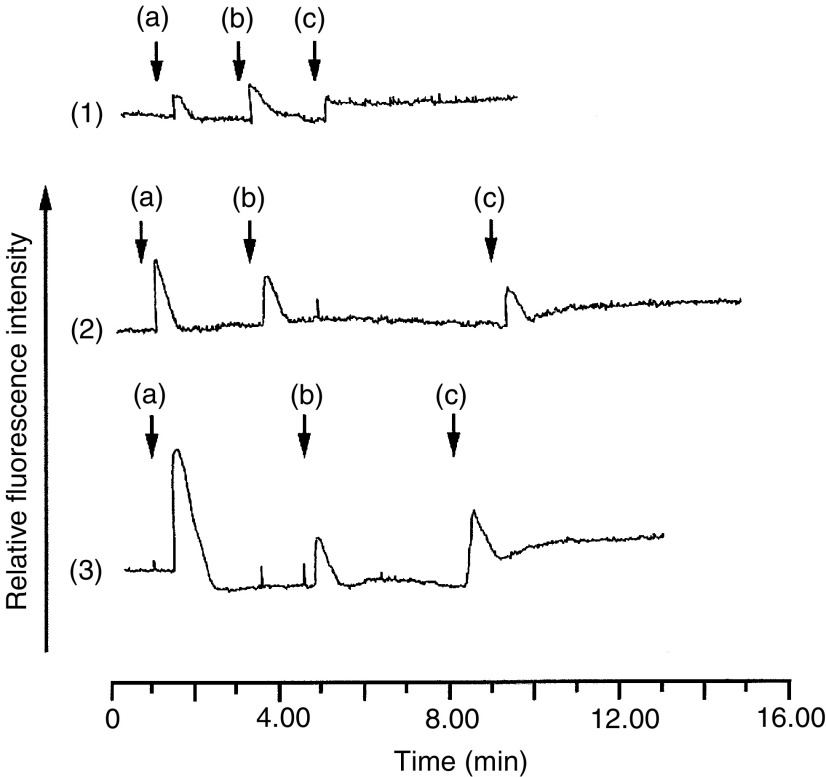
Intracellular calcium responses of (**1**) HBL (**2**) A375-SM and (**3**) C8161 melanoma cells to *α*-MSH+PIA (10 *μ*M). Cells were loaded with Fura-2 AM and the intracellular calcium release was detected by optical fluorimetry. +(−)-*N*^6^-(2-phenylisopropyl)-adenosine was always added at the same time as *α*-MSH indicated by position of individual vertical arrows. (a) *α*-MSH (10^−11^ M); (b) *α*-MSH (10^−9^ M) and (c) *α*-MSH (10^−7^ M).

The human HBL and murine B16F10-C1 melanoma cell line (used as a positive control) responded to *α*-MSH concentrations from 10^−13^ to 10^−6^ M with increasing cAMP (Figure 3A and B

). The C8161 and A375-SM melanoma cells did not respond to any concentrations of *α*-MSH with a detectable level of either extracellular or intracellular cAMP (Figure 3A and 3B). However, HBL (Figure 4 (1)

), A375-SM (Figure 4 (2)) and C8161 (Figure 4 (3)) melanoma cells all responded with increases in intracellular calcium to *α*-MSH+PIA addition at concentrations of *α*-MSH from 10^−13^ M to 10^−6^ M. Increases in intracellular calcium were only ever observed in the presence of 10 *μ*M PIA (a pharmacological adenosine agonist that inhibits any elevation of cAMP). (+(−)-*N*^6^-(2-phenylisopropyl)-adenosine alone did not elevate calcium in any of the cell lines (results not shown). It was observed that the magnitude of the response obtained for the C8161 melanoma line (Figure 4 (3)) was consistently greater than that observed for the HBL or A375-SM lines (Figure 4 (1 or 2)). A summary of intracellular calcium responses is given in Table 1

**Table 1:** Summary of the number of observed positive responses to *α*-MSH+PIA (10 *μ*M) in inducing an intracellular calcium signal in HBL, A375-SM and C8161 melanoma cells

	**Number of coverslips responding with an increase in calcium**		
**α-MSH (M)+PIA (10 *μ*M)**	**HBL**	**A375-SM**	**C8161**
10^−13^ M	3/6	6/6	2/2
10^−12^ M	5/6	6/6	1/1
10^−11^ M	9/9	7/7	4/4
10^−10^ M	6/6	6/6	3/3
10^−9^ M	6/6	6/6	5/5
10^−8^ M	6/6	6/6	5/5
10^−7^ M	6/6	7/7	4/4
10^−6^ M	6/6	10/10	6/6

.

### *α*-Melanocyte-simulating hormone and the invasion of melanoma cells through human fibronectin

As the three melanoma cell lines invaded through the fibronectin-Transwell™ layer at different rates, the invasion assay incubation time was adjusted to obtain a reproducible number of cells invading under basal control conditions, equivalent to between 20 and 50% of total cell number. The C8161 melanoma cell line was highly invasive with 49±6.8% of the total cell population invading after 15 h (*n*=5). In contrast, the HBL melanoma cell line gave invasion of 42±2% (*n*=10) after 20 h and A375-SM cells were the least invasive with 20±2.6% of the total cell population invading after 52 h (*n*=7).

**Figure 5:**
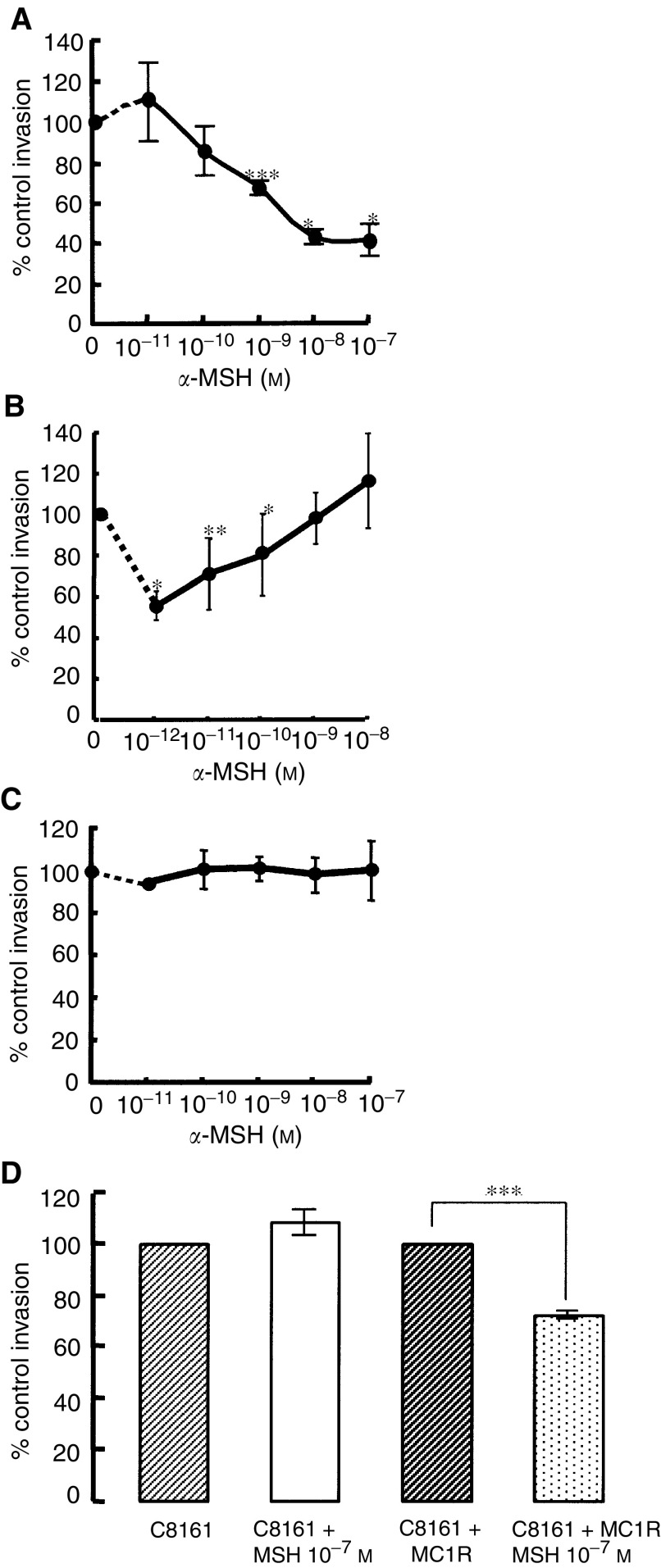
Effect of *α*-MSH on melanoma invasion through human fibronectin. The invasion assay was run for (**A**) 20 h with HBL melanoma cells, (**B**) 52 h with A375-SM cells, (**C**) 15 h with C8161 cells and (**D**) 15 h with untransfected and stably transfected (with MC-1R) C8161 cells. Values shown represent the raw data (% mean invasion) obtained for each experiment, which were normalised by relating all values to the control level of invasion in each individual experiment (taken as 100%). Statistical comparison is made between invasion in the absence (control) and presence of *α*-MSH. ^*^P<0.05, ^**^P<0.01, ^***^P<0.001.

Using the above experimental conditions, *α*-MSH significantly inhibited invasion of the HBL melanoma cells through fibronectin (results are presented as the mean percentage of invasion found in the control), in a concentration-dependent manner (Figure 5A

). A maximum inhibition of 58% for HBL cells was observed using 10^−7^ M *α*-MSH. For A375-SM melanoma cells, relatively low concentrations of *α*-MSH (10^−12^–10^−10^ M) were observed to significantly inhibit invasion with 10^−12^ M, demonstrating 46% inhibition (Figure 5B). Interestingly, *α*-MSH concentrations above 10^−9^ M were ineffective in inhibiting invasion of this cell line. In contrast, *α*-MSH had no effect on the invasive ability of C8161 cells through human fibronectin (Figure 5C). However, when these cells were stably transfected with the wild-type MC-1R, *α*-MSH (at 10^−7^ M) inhibited invasion by approximately 30% (72±1.9%, *n*=4, *P*<0.0006, Figure 5D). With the HBL melanoma cells, the inhibitory effect of *α*-MSH was confirmed to be via a cAMP mechanism by using the receptor-independent agents, IBMX and forskolin. Table 2

**Table 2:** Summary of the effect of *α*-MSH, IBMX, forskolin on the invasion of melanoma cells through human fibronectin

		**% Control invasion (mean+s.e.m. (*n*))**			
**Agent**	**Concentration (M)**	**HBL**	**A375SM**	**C8161**	
Control	—	100±4.8 (10)	100±13 (7)	100±13.9 (5)	
*α*-MSH	1 × 10^−12^	—	54±3.6 (5)*	—	
	1 × 10^−11^	110±19.1 (3)	77±9.7 (7)***	94±1.5 (3)	
	1 × 10^−10^	86±12.1 (4)	84±10.8 (7)*	101±9 (3)	
	1 × 10^−9^	65±2.3 (13)***	99±8.3 (6)	101±5.5 (3)	
	1 × 10^−8^	44±2.5 (3)*	109±15.2 (6)	98±8.1 (3)	
	1 × 10^−7^	42±7.9 (3)*	—	100±14.3 (3)	
IBMX	1 × 10^−5^	98±7.5 (3)	—	—	
	5 × 10^−5^	73±7.1 (7)*	108±6.9 (3)	—	
	1 × 10^−4^	55±4 (7)***	64±13.1 (3)	—	
	2.5 × 10^−4^	45±11.7 (3)	77±9.7 (3)	—	
	5 × 10^−5^	30±5.8 (3)*	—	—	
Forskolin	1 × 10^−6^	113±6.8 (3)	—	109±6 (3)	
	1 × 10^−5^	102±10 (6)	61±10.1 (3)	79±5 (3)*	
	1 × 10^−4^	41±3.2 (6)***	109±2.8 (3)	47±6 (3)**	

Statistical comparisonis made between invasion in the absence (control) and presence of pharmacological agent.

* *P*<0.05,

** *P*<0.01,

*** *P*<0.001.

shows that IBMX (at 5 × 10^−4^ M) significantly inhibited HBL melanoma cell invasion by 70% and forskolin (at 1 × 10^−4^ M) inhibited invasion by 59%. For A375-SM cells, the largest inhibition of invasion observed was with 1 × 10^−4^ M IBMX (36%) or 1 × 10^−5^ M forskolin (39%), but neither results were statistically significant. A cAMP mechanism was also confirmed for the C8161 melanoma cell line, as cell invasion was inhibited by 1 × 10^−5^ M (21%) and 1 × 10^−6^ M (53%) forskolin. Furthermore, combination of *α*-MSH (1 × 10^−9^ M) and IBMX (5 × 10^−5^ and 1 × 10^−4^ M) with HBL melanoma cells resulted in a further significant reduction in HBL invasion, compared to either *α*-MSH or IBMX alone (Table 3

**Table 3:** Summary of the effect of *α*-MSH, IBMX, forskolin and H89 on the invasion of HBL melanoma cells through human fibronectin

**Agent(s)**	**% Control invasion of HBL cells (mean±s.e.m. (*n*))**
Control	100±4.8 (13)
*α*-MSH (10^–9^ M)	65±2.3 (13)***
IBMX (5 × 10^−5^ M)	73±7.1 (7)*
IBMX (10^−4^ M)	55±4 (7)***
α-MSH (10^−9^ M)+IBMX (5 × 10^−5^ M)	48±7.4 (3)*
α-MSH (10^−9^ M)+IBMX (10^−4^ M)	35±5.9 (4)***
Forskolin (10^−5^ M)	102±10 (6)
Forskolin (10^−4^ M)	41±3.2 (6)***
*α*-MSH (10^−9^ M)+forskolin (10^−5^ M)	101±15.9 (3)
*α*-MSH (10^−9^ M)+forskolin (10^−4^ M)	39±2.2 (3)**

Statistical comparison is made between invasion in the absence (control) and presence of pharmacological agent.

* *P*<0.05,

** *P*<0.01,

*** *P*<0.001

). However, no further reduction in invasion was observed when forskolin (1 × 10^−4^ M) was combined with *α*-MSH (1 × 10^−9^ M), in comparison to either agent alone.

### *α*-Melanocyte-simulating hormone and melanoma cell invasion through reconstructed skin

**Figure 6:**
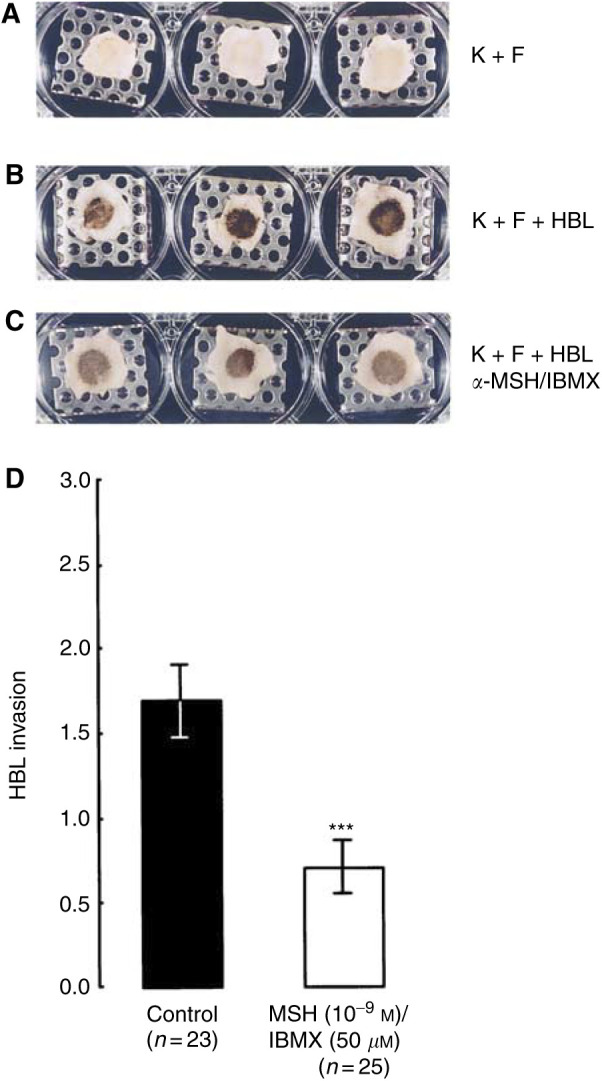
Melanoma cell invasion through reconstructed skin. Morphology (**A–C**) of composites and the extent of HBL invasion (**D**) through reconstructed skin±*α*-MSH/IBMX. In (**A**) composites contain keratinocytes (**K**) and fibroblast (**F**) cells; in (**B**) composites contain keratinocytes, fibroblasts and HBL melanoma cells, and in (**C**) composites contain keratinocytes, fibroblasts, HBL melanoma cells and *α*-MSH/IBMX. Statistical comparison in (**D**) is made between HBL cell invasion in the absence (control) and presence of *α*-MSH/IBMX. ^***^P<0.001.

A more clinically relevant model of human reconstructed skin (detailed in Eves *et al*, 2000) was used to investigate the actions of *α*-MSH on invasion of the HBL melanoma cell line. The other two cell lines were not investigated using this model, as they had shown limited (A375SM) or no (C8161) response to *α*-MSH in the fibronectin invasion model. A midrange inhibitory concentration of 1 × 10^−9^ M *α*-MSH was used. In order to potentiate *α*-MSH in this model, it was also combined with 5 × 10^−5^ M IBMX. Addition of *α*-MSH/IBMX had no obvious effect on the pigmentation of the HBL cells (Figure 6A–C

) in this skin model and did not affect the gross morphology of the composites; however, it did affect HBL dendricity (results not shown).

**Figure 7:**
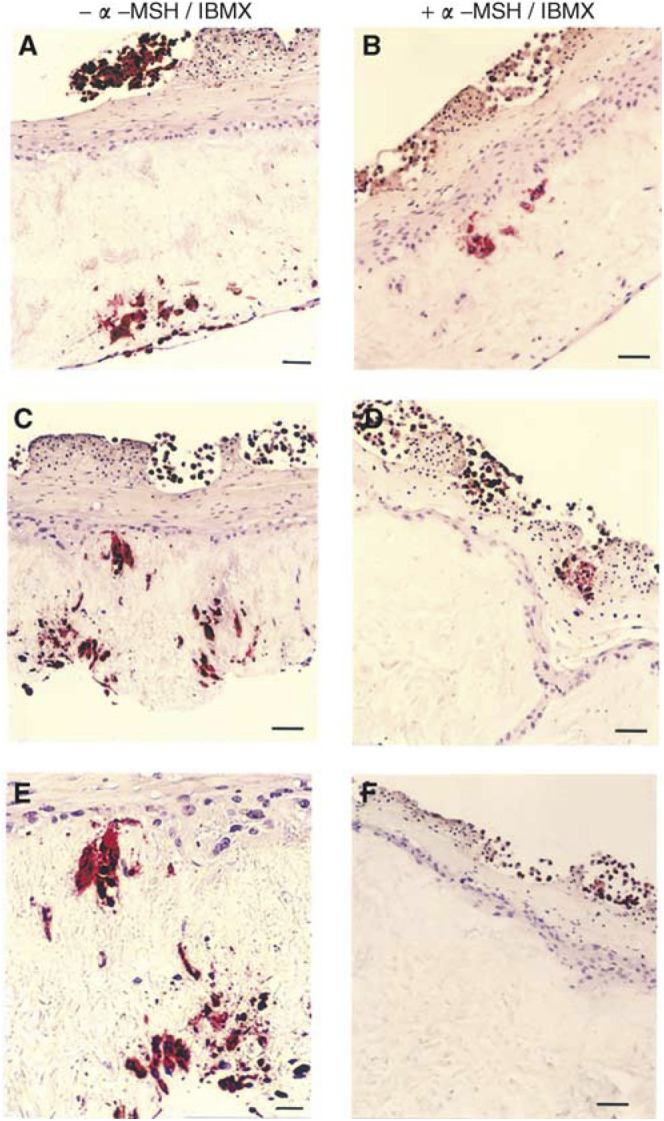
Histology of melanoma invasion through reconstructed skin composites containing keratinocytes, fibroblasts and HBL cells±*α*-MSH/IBMX. Composites were cultured for 14 days in the absence (**A**, **C** and **E**) and presence (**B**, **D** and **F**) of *α*-MSH (1 × 10^−9^ M)/IBMX (50 *μ*M). Composites have been labelled for HMB45 (for detection of HBL cells). Bar for (**A**)–(**F**)=40 *μ*m; bar for (**E**)=20 *μ*m.

After a 2-week culture period where the composites were routinely fed with fresh media containing *α*-MSH (1 × 10^−9^ M) and IBMX (5 × 10^−5^ M) every 3–4 days, they were sectioned and immunohistochemically labelled with an antibody to HMB45. Visual analysis of these sections clearly demonstrated that compared to control composites (Figure 7A,C,E

), *α*-MSH/IBMX (Figure 7B,D,F) had inhibited HBL invasion into the dermis. Semiquantitative analysis (see Figure 6D) was used to assess the extent of HBL invasion in the presence and absence of *α*-MSH/IBMX. Extensive invasion in the absence of *α*-MSH/IBMX was observed in 22 out of 23 composites, while invasion in the presence of *α*-MSH/IBMX was only observed in 15 out of 25 composites. The overall inhibition was 59% (*P*<0.0005).

### *α*-Melanocyte-simulating hormone and cytokine-induced activation of NF*κ*B/p65 in melanoma cells

**Figure 8:**
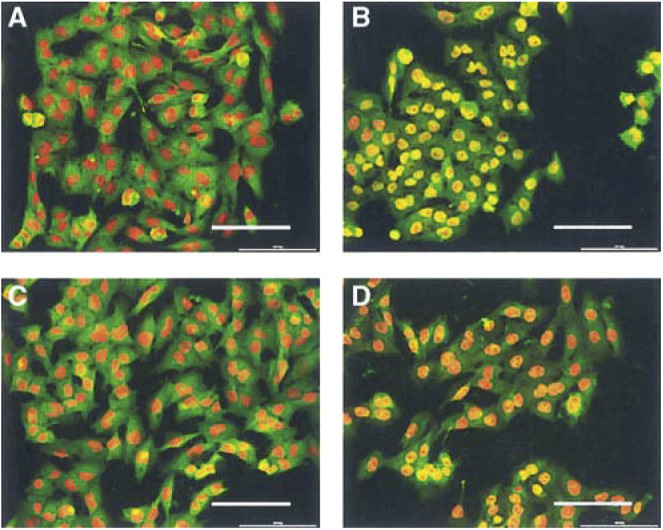
A375-SM melanoma cells immunolabelled for the p65 subunit of the NF-*κ*B complex. The p65 subunit (green fluorescence) was visualised using epifluorescent illumination (FITC filter, *λ*_ex_=490 nm, *λ*_em_=523 nm), while nuclei (red fluorescence) were visualised with a rhodamine filter (*λ*_ex_=555 nm, *λ*_em_=580 nm). Cells were either left untreated (**A**), stimulated with TNF-*α* (300 U ml^−1^, 60 min) (**B**), treated with *α*-MSH (10^−9^ M) (**C**) alone, or pretreated with *α*-MSH (10^−9^ M, 15 min) followed by stimulation with TNF-*α* (300 U ml^−1^, 60 min) (**D**). Activation of NF-*κ*B is observed as translocation to the nucleus (TNF-*α* alone, **B**) and inhibition by *α*-MSH+TNF-*α* as retention in the cytoplasm (**D**). Bar=100 *μ*m.

**Figure 9:**
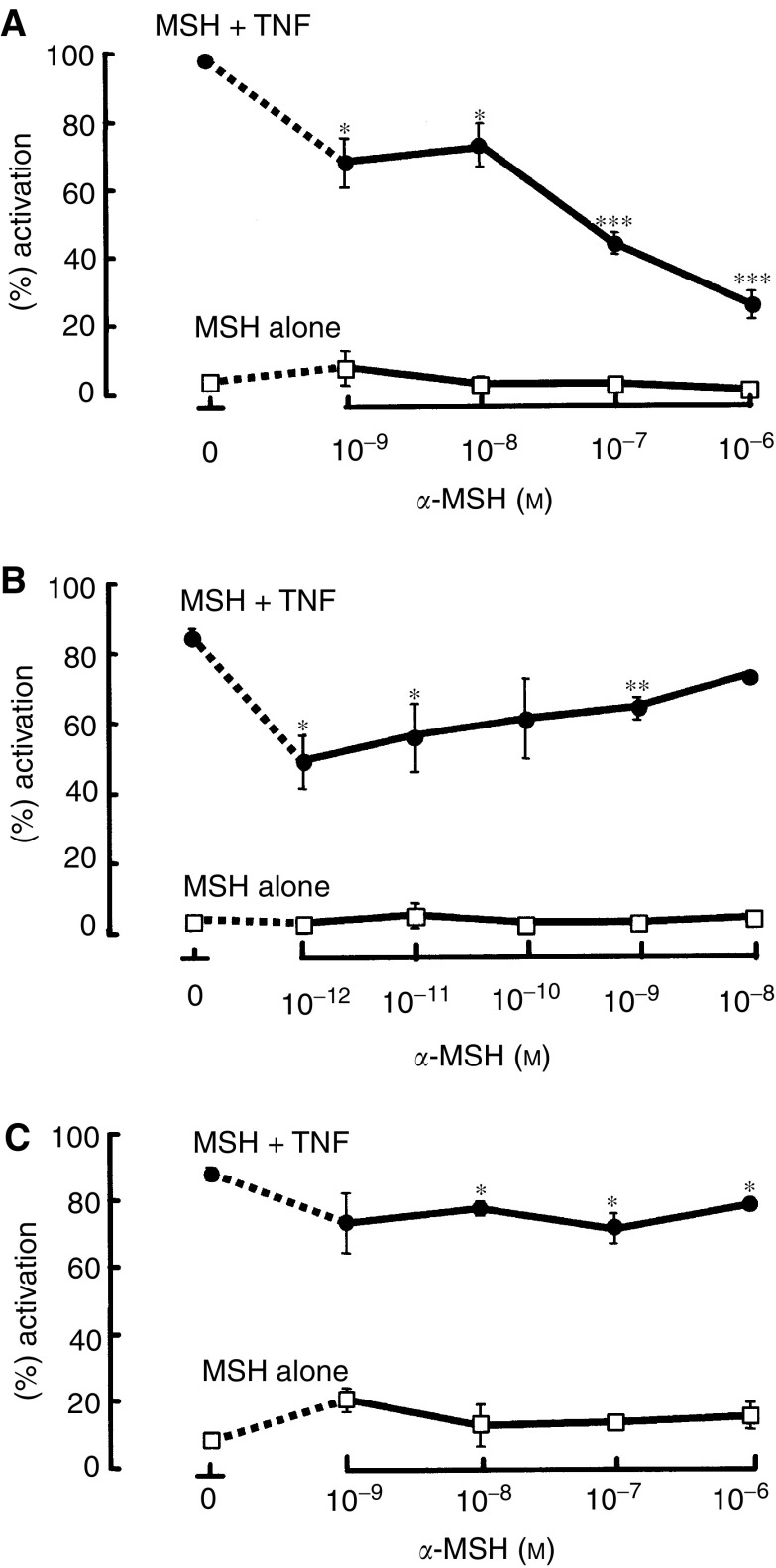
Effect of *α*-MSH on TNF-*α* stimulated NF-*κ*B activity in (**A**) HBL, (**B**) A375-SM and (**C**) C8161 melanoma cells. Statistical comparison is made between cells treated with TNF-*α* alone and cells pretreated with *α*-MSH followed by stimulation with TNF-*α*. ^*^P<0.05, ^**^P<0.01, ^***^P<0.001.

NF-*κ*B translocation from the cytoplasm into the nucleus was observed for all melanoma lines (HBL, A375-SM, C8161) in response to stimulation with TNF-*α* (60 min; shown for A375-SM, Figure 8

). Control untreated melanoma cells showed no evidence of NF-*κ*B activity (Figure 8A). After 1-h incubation with TNF-*α* (300 U ml^−1^), 85% of A375-SM cells showed an increase in activation (Figures 8B and 9B

). Addition of *α*-MSH alone (10^−12^–10^−8^ M) had no effect (Figure 8C and 9B); however; a 15 min preincubation (10^−12^–10^−8^ M) followed by a 1 h stimulation with TNF-*α* (300 U ml^−1^) resulted in a significant, concentration-dependent inhibition of TNF-*α*-induced NF*κ*B/p65 activation (Figures 8D and 9B). HBL cells displayed 97% NF-*κ*B activation in response to TNF-*α*. Addition of *α*-MSH alone (10^−9^10^−6^ M) had no effect, but preincubation with *α*-MSH (10^−9^–10^−6^ M) for 15 min followed by a 1 h stimulation with TNF-*α* (300 U ml^−1^) resulted in a concentration-dependent inhibition of TNF-*α* stimulated activity (Figure 9A). C8161 cells also had inactive NF-*κ*B in the absence of stimulators. Treatment with TNF-*α* (300 U ml^−1^) for 1 h activated NF-*κ*B to 88% of cells (Figure 9C). Addition of *α*-MSH alone (10^−9^ M to 10^−6^ M) resulted in a very slight but not significant increase in NF-*κ*B activity (Figure 9C). Preincubation of the C8161 cell line with *α*-MSH (1 × 10^−9^–1 × 10^−6^ M) followed by a 1 h stimulation with TNF-*α* (300 U ml^−1^) only inhibited NF*κ*B/p65 activation by around 10–15%. This was demonstrated to be marginally significant (*P*<0.05), but compares poorly to the 70% inhibition of *α*-MSH on HBL cells, and 35% inhibition on A375-SM cells.

## DISCUSSION

The aim of this study was to find out more about the role of *α*-MSH in influencing melanoma progression. We compared three genetically distinct human melanoma cell lines and examined to what extent *α*-MSH influenced cell invasion through a fibronectin protein layer and (for one of the lines) through a reconstructed skin model. The results obtained were compared with the ability of *α*-MSH to inhibit proinflammatory cytokine TNF-*α* activation of NF-*κ*B, elevate cAMP and stimulate intracellular calcium. The main findings of this study were that while all three lines expressed MC-1R, they differed markedly in their response to *α*-MSH. For HBL cells, which express wild-type MC1R, *α*-MSH elevated intracellular and extracellular cAMP, induced an increase in intracellular calcium, was very effective at inhibiting invasion of cells through fibronectin and through a reconstructed skin model and also attenuated the response of the cells to TNF-*α*. The A375-SM cells were homozygous for Arg151Cys for the MC-1R, and responded partially in that *α*-MSH inhibited invasion and the response to TNF-*α* at low *α*-MSH concentrations, but with negligible responses at higher concentrations. *α*-Melanocyte-simulating hormone induced intracellular calcium levels but did not elevate intracellular or extracellular cAMP to detectable levels. C8161 cells were heterozygous Arg151Cys for MC-1R, and were unresponsive to *α*-MSH with inhibiting invasion, had a small inhibitory response to TNF-*α* and failed to elevate intracellular or extracellular cAMP (although intracellular calcium responses were detected). However, stable transfection of these cells with the wild-type MC-1R showed that these cells were then able to respond to *α*-MSH with a significant reduction in invasion.

The HBL melanoma cell line has previously been demonstrated to express 1000–3000 MSH receptor-binding sites per cell (Ghanem *et al*, 1988), and A375 and C8161 melanoma cell lines have also been demonstrated to have MSH receptors (Baumann *et al*, 1997) and MSH binding sites (Sharma *et al*, 1996). In comparison, normal human cutaneous melanocytes are reported to express between 300 and 1000 binding sites per cell (Donatien *et al*, 1992; De Luca *et al*, 1993). In the present study, MC-1 receptors were identified on the cells by immunofluorescent microscopy, Western blotting and MSH binding sites using radiolabelled *α*-MSH. In addition, we also sequenced both alleles of the MC-1 receptor genomic DNA. Western blotting identified the presence of the MC-1 receptor at a molecular weight of 35 kDa, corresponding to the predicted molecular weight based on DNA sequencing data. As previously reported, binding studies confirmed that HBL melanoma cells had a relatively high receptor number. C8161 cells had a number approximating that found in normal skin melanocytes, while A375-SM cells had a relatively low number of receptors. However, all cells displayed similar receptor binding affinity for *α*-MSH. (It was interesting to note that the HBL and A375-SM cell lines were also positive for the MC-2R, in contrast to the C8161 melanoma line.) Melanocortin-1 receptor DNA sequencing showed that the HBL line expressed wild-type MC-1R, whereas the A375-SM and C8161 lines were homozygous and heterozygous for Arg151Cys, respectively. This polymorphism is known to convey a loss of function on receptor coupling to cAMP (reviewed in Wikberg *et al*, 2000). The heterozygous expression of Arg151Cys would suggest a potential for signalling; however, partially functional responses in the A375-SM line (homozygous Arg151Cys) would suggest either that this polymorphism conveys some function (as opposed to total loss) or that a signal other than cAMP is conveyed from the receptor in a region outside of the Arg151Cys site.

A number of polymorphic studies of the MC receptor are related to incidence of melanoma (Box *et al*, 1997; Jiménez-Cervantes *et al*, 2001). However, not all studies are in agreement (Ichii-Jones *et al*, 1998). *α*-Melanocyte-simulating hormone has been demonstrated to affect melanoma growth and attachment to ECM proteins in cells with wild-type MC-1, but not in cells transfected with polymorphic variants (Robinson and Healy, 2002). The current study provides strong evidence to suggest that two out of three human cell lines that possessed MC-1R polymorphisms, which gave a loss of cAMP response to MSH, resulted in cells that failed to respond to MSH with any reduction of invasion or protection against proinflammatory cytokine attack. Stable transfection of the C8161 cell line with the wild-type MC-1R and introduction of sensitivity to *α*-MSH provides possibly the strongest evidence yet that this receptor is involved in melanoma invasiveness.

As previously stated, there is controversy concerning the role *α*-MSH in the progression of melanoma. Most of the experimental studies have been carried out on murine melanoma cell lines, known to express between 3000 and 10 000 MSH binding sites per cell (Lambert and Lerner, 1983; Siegrist *et al*, 1989; Lunec *et al*, 1993), while human melanoma cells only express around 1000–3000 binding sites per cell (Ghanem *et al*, 1988). It is difficult to make firm conclusions concerning the role of *α*-MSH in human melanoma progression from murine studies. The inhibitory effect of *α*-MSH on the HBL cell line is similar to that reported previously (Murata *et al*, 1997) for highly invasive murine B16-BL6 melanoma cells. Melanoma invasion of the three cell lines was also inhibited (achieving significance for two out of three cell lines) by forskolin and/or IBMX (agents which elevate cAMP), supporting the idea that melanoma invasion is under the influence of adenylate cyclase/cAMP post-receptor signalling. This is consistent with earlier investigations showing a positive correlation between cAMP responsiveness of murine melanoma cell lines *in vitro* and the metastatic success of these lines *in vivo* (Sheppard *et al*, 1986; Hill *et al*, 1990).

The use of a reconstructed human skin model is particularly useful for studying melanoma cell invasion, as it provides a physiologically relevant dermal and epidermal structure (Eves *et al*, 2000). The melanoma cells are physically proximal to human keratinocytes and fibroblasts contained within an architecture that very closely approximates that of native skin tissue. The model also has a basement membrane structure, which is highly relevant to melanoma metastasis. Using this model we found that *α*-MSH (at 10^−9^ M)/IBMX (5 × 10^−5^ M) inhibited HBL melanoma cell invasion by 59%.

*α*-Melanocyte-simulating hormone can inhibit TNF-*α*-stimulated NF-*κ*B activity in human melanocytes, melanoma cells and keratinocytes (Haycock *et al*, 1999, 2000; Moustafa *et al*, 2002). In all of these cells, *α*-MSH inhibited the response to TNF-*α* by around 50%. In the current study, TNF-*α* activated NF*κ*B/p65 in over 80% of all melanoma cells within 60 min. The HBL melanoma line responded to *α*-MSH with an inhibition of NF-*κ*B. The A375-SM melanoma line responded to low *α*-MSH concentrations with an inhibition of NF-*κ*B, whereas higher *α*-MSH concentrations were ineffective. In contrast, C8161 cells were largely unresponsive to any *α*-MSH concentrations.

The correlation observed between the anti-invasive response of the melanoma lines and the anti-inflammatory response to *α*-MSH was very strong. We have shown previously that the anti-inflammatory response to *α*-MSH is also mediated via cAMP, suggesting a strong correlation between anti-invasive and anti-inflammatory responses. HBL melanoma cells responded to *α*-MSH in a concentration-dependant manner with elevation in intracellular and extracellular cAMP (as did the murine B16 F10C1 melanoma line, used as a positive control). However, the A375-SM and C8161 lines had no detectable increases in cAMP. The lack of an *α*-MSH response observed for the C8161 cells and a partial response for the A375-SM cells are consistent with the lack of a cAMP signal in these cells.

In contrast, all cell lines responded to *α*-MSH with acute elevations in intracellular calcium. Dual signalling from the MC receptor is not a new concept. In 1984, *α*-MSH-stimulated adenylate cyclase was reported to be regulated by calcium and calmodulin in a biphasic manner. Submicromolar concentrations of calcium were shown to be required for optimal *α*-MSH-stimulated cAMP production; slightly higher micromolar concentrations led to inhibition of adenylate cyclase (Mac Neil *et al*, 1984) and activation of calcium/calmodulin phosphodiesterase, resulting in cAMP breakdown in murine melanoma cells. In these same cells, *α*-MSH was also demonstrated to activate protein kinase C (Buffey *et al*, 1992). Although *α*-MSH may activate the phospholipase C signalling pathway (resulting in increased inositol phosphates, intracellular calcium and activation of protein kinase C) in addition to activating the adenylate cyclase cAMP signalling pathway, the overwhelming evidence from the literature (as reviewed in Eberle, 1988) points to the majority of the actions of *α*-MSH being mediated by cAMP. The relevance of the dual signalling remains unclear although the calcium signal can modify the cAMP-mediated actions of *α*-MSH on melanogenesis (Buffey *et al*, 1992). The simplest hypothesis to explain dual signalling is that most actions of the *α*-MSH-induced phospholipase C signalling are explained by it suppressing the *α*-MSH-induced cAMP signal (as proposed in Mac Neil *et al*, 1984). There is nothing in the current study to contradict this hypothesis as all of the functional biology of *α*-MSH that we observed could be mimicked by elevating cAMP. The two cell lines that failed to generate a cAMP signal to *α*-MSH did give a calcium signal, indicating that the receptors were adequately coupled to generate calcium but not cAMP.

The data obtained in this study highlight an interesting question regarding MC receptor expression and the functional activity of *α*-MSH in melanoma cells. While immunofluorescent microscopy demonstrated that all three melanoma lines express MC-1 receptors, *α*-MSH binding studies showed that only HBL cells have a high number of MSH receptors capable of generating a cAMP signal. Melanocortin-1 receptor polymorphisms have been associated with red hair and fair skin, and also with cutaneous melanoma in humans. Hence, it is of considerable interest that two of the lines investigated in this study contained homozygous and heterozygous polymorphisms, respectively. The Arg151Cys polymorphism, along with others (Arg160Trp and Arg294His) results in loss of receptor function, with association of an increased risk for melanoma (reviewed in Xia *et al*, 1995). The importance of receptor polymorphisms is further highlighted by the fact that when C8161 cells were stably transfected with the wild-type MC-1R, the cells then responded with a significant inhibition of invasion in response to *α*-MSH.

Melanoma cells that possess sufficient functional MC receptors may therefore respond to endogenous or exogenous *α*-MSH with a reduction in their ability to invade through ECM proteins, and ability to resist proinflammatory cytokines. The former properties would tend to reduce melanoma invasion. Equally, it might be argued that resisting proinflammatory cytokine action is consistent with promoting melanoma invasion by assisting melanoma cells to escape immune surveillance. It is difficult to conclude how important the anti-invasive actions of *α*-MSH *in vitro* are *vs* the protective (and hence proinvasive) activity in preventing immune detection. We speculate that *α*-MSH may retard melanoma escape from the primary tumour on the one hand (and initial metastasis), and on the other may promote melanoma invasion by reducing the response of cells to proinflammatory cytokines. Thus, a simple hypothesis to explain our present data would be that *α*-MSH initially acts to reduce early tumour spread, but affords protection to those cells that do escape from a primary tumour site.

A major biological function of *α*-MSH in the melanocyte, probably retained in the melanoma cell, may be the protection of cells from proinflammatory cytokines and oxidative stress. A common response of cells to such stress includes upregulation of adhesion molecules, some of which would normally bring cells to the attention of the immune system. Adhesion molecules such as integrins may also be upregulated and promote melanoma interaction with ECM proteins in terms of migration and invasion through the ECM. We have recently shown that while the proinflammatory cytokine TNF-*α* upregulates integrin (*α*3, *α*4, *β*1) expression in HBL melanoma cells, treatment with *α*-MSH results in a reduction in integrin expression (Zhu *et al*, 2002). These results suggest that *α*-MSH can effectively reduce cytokine-induced upregulation of adhesion molecules such as integrins that would tend to increase melanoma invasiveness through ECM proteins while also reducing expression of adhesion molecules that promote interaction between melanoma cells and the immune system (e.g. ICAM-1 as we previously demonstrated (Hedley *et al*, 1998b; Morandini *et al*, 1998)), thus enhancing melanoma escape of immune surveillance.

In conclusion, this study suggests that *α*-MSH has the potential to retard metastatic spread but also to reduce the ability of the immune system to detect tumour cells, in some melanoma cells. Both responses to *α*-MSH appear to be linked to the cAMP signalling pathway, but clearly the downstream signalling from the MC-1R is complex and far from resolved. As metastatic melanoma remains an extremely challenging cancer to treat, we suggest the role of *α*-MSH in melanoma progression merits further investigation as it may hold clues to the metastatic success of the tumour and may offer new approaches to developing therapies to reduce metastatic spread or enhance the ability of these cells to be detected by the immune system.
